# Odontological Society of Pennsylvania—Tenth Anniversary Meeting

**Published:** 1889-03

**Authors:** 


### ODONTOLOGICAL SOCIETY OF PENNSYLVANIA.

### TENTH ANNIVERSARY MEETING.

The Tenth Anniversary of the Odontological Society of Pennsylvania was held at Association Hall, Philadelphia, Dec. 12th and 13th, 1888.

*Wednesday, December 12th.*

The meeting was called to order at 2.30 P. M., by the President, Dr. Edward C. Kirk.

Prayer by Wayland Hoyt, D.D.

An address of welcome was made by Prof. Charles J. Essig, of Philadelphia, one of the charter members of the society.

*Gentlemen:*—It becomes my duty, as it is my great pleasure, to extend to the guests of the Odontological Society of Pennsylvania, a sincere welcome.

It seems fitting that we should thus celebrate the tenth anniversary of this society for the importance of the work of a decade of activity and usefulness, of such an organization, cannot well be overestimated.

To say that it is an occasion for congratulation would but feebly express the sentiments of those who have watched its growth from its foundation.

Inaugurated at a period that marked a distinct impulse in dental affairs, it did not escape the opposition that usually greets such enterprises; but its ten years of successful growth abundantly show that at its organization it met an existing want.

A society like the Odontological is a vehicle by which, through the reading of valuable papers, discussions, criticisms, clinics and demonstrations, a large amount of practical information reaches the profession at large.

The progress of dental art and science would of course be more rapid, if it were possible for us at a glance to recognize and separate the merely speculative from the practical; but amongst the methods and theories brought before our societies at their monthly meetings, the two are often nearly equally represented.

"It has ever been the practice of mankind to let the wheat and the tares grow, and bring forth their fruit together," and it is a healthy sign when men organize, and meet regularly for the purpose of garnering the one and rejecting the other, or stirred by a zeal to be of use in their day and generation, to freely impart the knowledge and experience which comes to them in their professional duties.

It emphasizes the difference between the past and the present, for the fraternal instinct, which leads men to seek intercourse of this kind and with this object, did not exist in the early days of our calling, or else it was held in abeyance by less worthy motives.

Free interchange of ideas and publicity, such as are afforded by the societies on the one hand, and the dental journals on the other, are essential to the development of a specialty, which draws from the arts and sciences, whatever in the practice of its various branches it finds useful.

In the earlier days of dentistry, valuable discoveries must have been made many times, without the profession acquiring thereby any new fact.

Indeed we are often reminded of the disposition of "those old fellows," "to steal our modern ideas," when some young member, consumed by a desire to achieve fame, describes his latest discovery, only to find that it had been done perhaps before he was born. Indeed, there is hardly a method of recent adoption of which this cannot be said. It is true of both bridge work and implantation, the two most important of the recent contributions to dentistry.

To prevent new discoveries from being either lost or too frequently invented, there must be just such a combination of favorable circumstances, as we have in our societies and journals.

Among the members of our profession will be found some of an inventive turn, and we may also have others equally useful though perhaps less praiseworthy, whom we shall designate as appropriators, and while we have not asked our visiting brethren here because, "having found out pretty thoroughly what each other knows," we want other fields and pastures new, yet Philadelphia has long been distinguished by extraordinary activity, in discovering the value of novel methods and appliances, so that I can assure you that anything new exhibited here will not be "wasted on the desert air."

But be this as it may, the fact remains that no matter in what way the new theory, device or fact, gets before the society, it becomes through its agency the property of the professional world, and humanity is benefitted thereby.

There are other directions besides the discussion of methods or scientific problems, and the general aim of preventing the intrusion of unsubstantial theories, in which our dental societies can be of great use. Their fundamental object being the general good of the profession, it would seem perfectly proper, and in keeping with its legitimate function to scrutinize the present system of dental education, providing it does so impartially and without fear or favor. It would not be true to its own interests, if it did not feel dissatisfied with any system which does not give promise of the highest possible attainment.

But, gentlemen, these are thoughts that naturally present themselves to one whose pleasant duty it is to open this anniversary session, with a cordial greeting to our guests who have joined us in the celebration of a decade of our existence, devoted to the interests of the profession to which we owe allegiance.

I presume the honor of extending a formal greeting to you fell to my lot, because I am not supposed to be given to lengthy speeches; indeed the large number of essays, discussions, and incidental work which will come before the society, forbid such encroachment upon the time, short enough at best, which has been reserved for the purpose.

Let us endeavor to make this meeting one of pleasure, as it surely must be in the opportunity to social intercoure which it offers, and of profit in the evidences of professional advancement which will doubltess be exhibited here, that our guests may carry home with them pleasant memories of the 12th and 13th of December, 1888.

*Dr. A. L. Northrop, New York*, made a very happy extemporaneous response but which, on account of the absence of the reporter, was not obtained.

*Evening Session, 8 o'clock, P. M.*

Lantern exhibit, illustrating Dental Histology and Pathology, by Drs. R. R. Andrews, Geo S. Allen, and W. X. Sudduth.

*Dr. Andrews* presented the subject of the development of the teeth, dwelling upon the formation of calco-globulin.

*Dr. Allan* showed photo-micrographs of artificial and natural decay, and the micro-organisms causing the destruction of tooth-substance.

*Dr. Sudduth* illustrated the subject of inflammation in non-vascular tissues, using photographs of a large number of specimens of pathological ivory.

DISCUSSION ON DR. ALLAN'S PAPER ON DENTAL CARIES READ AT THE AFTERNOON SESSION, AND THE EXHIBIT OF THE EVENING.

*Dr. W. A. Atkinson, New York City*—I rejoice in the progress that has been made; but my joy is taken away when I see assumption of wisdom take the place of facts. It is difficult to speak of the specimens, for there are only a few in an audience like this who are acquainted with them. Those who have never done this work and developed such specimens are unable to know from the screen the beauty and excellence of the work, even when it is well presented. The weakness of mankind is to pronounce matters as finalities when their investigations have not fully settled the subject. If we but hold ourselves within limitations of what we do see and know, and discriminate against that which is uncertain, we would make more sound progress.

Now it was said by Dr. Sudduth that there was no blood in the cornea. There is more blood than named in text books. He also said that the lymph spaces in the cornea under the inflammatory process became enlarged, until red blood corpuscles could pass into them and give the appearance so often seen in conjunctivitis. How do they get into the lymph spaces? Do they come from the main tract or from a capillary? I put this to persons who want to get at the solid truth with regard to inflammatory action.

To go back to the time when the specimen first shown, the left superior lateral incisor with a depression on its lingual surface and a small cavity in the emamel was cut, from that time I know George S. Allen has done excellent work. He cut that for me in Cleveland, O., about 1855 or 1856. We have grown a good deal in our work, but we stand exactly where Richard Owen stood, producing appearances that we cannot interpret. I want to develop that sort of investigation that knows no compromise until the truth is revealed.

*Dr. C. N. Pierce, Phila.*—So fully am I in accord with the paper read by Dr. Allan, that I have little pleasure in any effort I may make to criticise it. But a remark made by him and repeated by Dr. Sudduth seems to leave an impression upon the audience which I would like to modify. The non-vascular condition of the dentine is spoken of so positively that its organic matter and circulating system is quite ignored, yet simple experiments will test the prominence of both. A tooth placed in chromic or other acid for but a limited time becomes decalcified, and consequently flexible; this, the organic element of the tooth which is left, maintains much of its original contour though minus its original density, from the loss of its lime salts. A freshly extracted tooth, weighed and then thoroughly dried, will have been found to have lost perceptibly in weight, and on being again immersed in a liquid much of this is regained. This experiment tests quite satisfactorily the permeability of this dense tissue, and again the changes which take place in the color of the teeth, both physiologically and pathologically in their nature, are still further evidence of its possibilities in this direction. If these organs are so readily permeated by a fluid, then why not their vitality, sensibility and density, all be more or less under the influence of a vital fluid furnished by or emanating from the vessels in the pulp and peridental membrane, vascular tissues which hold the teeth in close connection with the arterial and nervous system. Does not every skilled operator recognize the varying sensibility of the dental tissues under different systemic conditions? Does he not also recognize a difference in density at different ages? While the language or tests of the books justify the expression, " non-vascularity of the dental tissues," yet accurately or critically speaking, it is in my judgment quite incorrect, and for the reason above illustrated that we much recognize vital and physiological changes which are constantly taking place during the life of the tooth. I know this has been doubted by some most excellent observers, but the evidence in my judgment is against them. In this most valuable paper Dr. Allan has given us beautiful illustrations of the etiology of dental caries, and in doing so has displayed influence or function of certain forms of bacteria. Now, while we recognize the harmful influence of these, and their essential presence in the progress of dental caries, we at the same time should not forget that there are innumerable other living forms or organisms in the oral cavity which are not only not harmful, but benign. They are *mess-mates* and not parasites in any sense of the term; among these are the lepthorthinx buccalis. Where there is pabulum organism will fine a habitat, and their multiplication and influence varies with their protection and food. When I think of the labor and expense bestowed by Drs. Allan, Andrews, and Sudduth in the preparation of these papers and specimens or illustrations—of the months and years of thorough and earnest effort, and of the result in illustrating so clearly the origin and the development of the teeth through their various progressive stages, from the simple homogeneous structure to the complex and heterogeneous one—I am proud that I am a dentist, and firm am I in my conviction that there is a future for dentistry; that we have as yet seen but the beginning, what is being but prophetic of what is to come.

But, Mr. Chairman, what is most sorrowful is that in spite of or in the face of all these beautiful illustrations in published text-books, we meet with statements long since exploded, statements begotten in ignorance and repeated and republished in the self-satisfied insufficiency of the author.

In this evening's work I have but one regret, and that is that with such opportunities for knowledge there should be standing room for another dentist in this house.

*Dr. R. R. Andrews, Cambridge, Mass.*—There are one or two points I would like to speak of, and although I shall have to differ somewhat from some here, I do so because I have reason for it.

Some years ago, there occurred here in Philadelphia a very pleasant meeting of a few who were interested in histology. I think

Drs. Black, Darby, Sudduth and Williams, among others, were in the room. I mentioned at that time the case of a tooth that came to me very frail, which I had filled with the old oxychloride of zinc, and in the course of two years I found instead of frail dentine it had become very hard. The tooth was entirely changed. I was much surprised to have Prof. Black tell me that there was no change in the structure of the dentine of the tooth. I know that the tooth was originally so soft that I could scrape off its substance with an excavator. The tooth was afterwards filled with gold, and Dr. Black told me at the time that if I should make a section of the tooth I should find that the tubuli were not calcified, but were filled with fatty globules.

We see superficial caries that have been stopped entirely in teeth of the first quality. There is a cavity, black or brown, that has been arrested by the reparatory action of the dentine. The color has been given by dead micro-organisms. I believe decay is arrested by some reparative action of the pulp. Instead of fat globules being found there, they are really globules of calcospherites, a first product of calcification. These tiny globules have been carried by some process into the tubuli of the dentine, and because of the life centre, the pulp, being so far from the periphery, it has not been able to carry out its full function.

I believe that in teeth of the first quality, where the decay has been stopped by natural processes, it has been stopped by the calcospherites being carried to the point of decay or injury, and resisting the attack of decay, perhaps the partial, if not full calcification of the tubuli at that point.

I think it is possible we may find in time that the white zone between the micro-organisms and the healthy dentine may not be partially decalcified, but may be a zone of resistance, I am by no means sure of this. Now, they say that the white zone may be seen in artificially produced decay. I have seen a few of Dr. Miller's specimens, but have never seen this softened zone. He places a piece of tooth into a culture, but the micro-organisms attack it from all directions, and I do not think that we can come to any positive conclusions that there is a white zone there.

There is certainly a certain amount of circulation through the tooth. There can be no doubt about it. It is not the same circulation as blood circulation, but there is a fluid and a certain circulation.

*Dr. W. H. Dwinelle, New York City*—Will not our memory, referring to the past, help us out of this difficulty? We know that teeth have had the dentine injected until they were quite red, after a severe accident, showing that under heavy pressure the blood corpuscles become broken down. We know the diameter of a blood corpuscle is about $\frac{1}{3000}$ of an inch, whereas the diameter of the tubuli are about $\frac{1}{10000}$, so that blood corpuscles could not enter into the tubuli. That being the case, the theory in olden times was, when endeavoring to account for the teeth becoming red, that the blood corpuscles were broken down, and became so fluid that it was capable of carrying its coloring matter with it. That was an old theory, and if it is a good one I do not see why it will not help us out on this question.

*Dr. James Truman, Phila.*—I was surprised to hear Dr. Sudduth make the statement that no dentine was formed except upon the odontoblastic layer. I question whether this assertion can be sustained, as it seems to me in confiict with known facts. There are several forms of dentine that by their development seem to antagonize the opinion that the formation of dentine is confined to the peripheral border of the pulp. The tubes in senile dentine are closed with a secondary formation, and are rendered translucent. In teeth thrown out of the jaw in earlier life the same appearance is frequently presented, the dentine exhibiting a perfectly transparent appearance, with an entire obliteration of the parietes of the tubes. Now what is the explanation of this? Moderate irritation will produce extra development of tissue, or, as I choose to formulate it, slight irritation produces extra development; excessive irritation, destruction. This is perfectly illustrated in the abrasion of the cutting edges of the anterior teeth. In slow caries the conditions for reformation are always present, and the transparent zone, in my opinion, is the result. The investigations of Wedl, Miller and others do not seem to me to give satisfactory results.

*Dr. G. S. Allan, New York City*—In reference to the point made by Dr. Pierce, I think we only differ on terms. It is quite difficult to make use of a definition that all understand alike. I think I agree with Dr. Pierce, and in the main also agree with Dr. Sudduth.

As to what Dr. Truman says about old teeth and secondary dentine, I will say that as far as I can understand the observations of Drs. Miller, Black and Sudduth, and from my own observations upon many teeth sections which I have in my cabinet, I do not believe that the tubules do become filled up with secondary dentine. I think Dr. Sudduth's explanation, while in the main theoretical, offers a very satisfactory solution to the question as to how the teeth change in character, appearance, and physical characteristics from youth to age, without the necessity of saying that there is an extra calcification. The filling up of the terminal ends of the dental tubules and canaliculi with a deposit of secondary dentine—granting such to be the case, would not, it seems to me, account for that extra hardness. There is not enough space in the tubuli to warrant that their filling up alone would account for this extra hardness and extra resistance to the instrument in cutting it.

I have quite a number of sections of teeth of old age, and one I will call attention to in closing. It is a section of a molar tooth from a person 60 years old. The section was made through the filling, and the tooth and the filling is shown in situ. It is an amalgam filling. There is no question in examining that tooth, that the tubules are all open, because where the presence of the mercury in the tubules is not evident, the presence of air is perfectly plain to be seen; that section I have in my possession, and to my mind it proves conclusively that the dental tubules do not become fiilled with a calcific deposit.

Subject passed.

---

DISCUSSION ON DR. PERRY'S PAPER ON THE TREATMENT OF PROXIMATE SURFACES.

*Dr. W. H. Dwinelle, New York City*--Certainly there is no one present who would desire the paper just read to be one sentence shorter. All familiar with my professional life know I have always advocated contour filling. We have simply fallen into the reasonable conclusion that Almighty God is the source of infinite wisdom, and when we mangle, deface or destroy these beautiful creations of his hand we take a step, at least, downward.

To be endorsed, as I have been so cordially on principles I advocated between thirty and forty years ago, is such a source of delight and comfort to me, and is such a complete fruition of my earlier hopes, that I am almost constrained to exclaim with the psalmist: "Now let thy servant depart in peace." The reason for contouring is patent to us all. The teeth should be restored to their pristine contour by bringing them to their largest circumference or diameter, so that they impinge at that point; and one reason for it is to prevent food from crowding up between the surfaces, as it otherwise would press upon the gums, to their injury. This is obviated by the teeth touching at their largest circumference like a row of oranges in contact. If this is not done, the teeth are often liable to leave their place in the arch, or retreat from their normal position.

The objection has been made to contour fillings in that we are not skillful enough to establish a perfect closure or joint at the cervical margin. Let us be more skillful, then. I can show you contour fillings I built up as far back as 1854; have seen them within a few days, and they are as perfect as if the operations were performed yesterday. The cervical point is where the greatest skill is necessary, for if it is imperfectly closed you will find it necessary to either remove the filling entire, or perform a very difficult operation to correct the mistake; failing here, you fail in everything.

In a treatise on crystal gold, which I published in the *American Journal of Dental Science* in 1855, I think all the arguments I could present at this time were presented then. I am not a prophet, but I have been congratulating myself that at that time I was inspired by something like the spirit of prophecy. I then advocated the restoration of the entire crowns of the teeth—molars as well as the rest—and this before the day of the rubber dam, too. How I succeeded is now a wonder to me. I used napkins, and had progressed so far in my system that I could stop off the flow from the buccal and sublingual ducts entirely. I made little tongs out of silver wire, with which I grasped the mouths of the ducts. I was able, by the use of these appliances, in conjunction with napkins, to stay off the flow of the saliva, so that when I took off the napkins I sometimes brought portions of the mucous membrane with them.

The dental engine is one of our greatest blessings, and yet I think it is doing a great deal of injury to the coming generation of our profession. A few years ago I read an article on the "Good and Bad of a Dental Engine." In the use of this appliance we lose the delicacy of touch and manipulation of the fingers so essential to the best work. Dr. Perry has incidentally alluded to it, and given you the note of warning, when he says that a young student, or rather young practitioner, is apt to lay aside the old instruments in excavating cavities and resort entirely to the engine. I am in hopes we shall reform in this respect.

*Dr. Chupein, Philadelphia*—Some eight or nine years ago, Prof. Flagg read a paper before the Odontological Society of New York, in which he made the following remark, relative to the two modes of operating on the teeth which were in vogue at that time: "'The Separationists' may be likened to the A B C of the profession, as Arthur, Bonwill, Chupein; while 'the Contourists' embrace the whole alphabet from A to W—Atkinson and Webb."

Now, while I was classed among the former division, I worked then in all earnestness, and endeavored with all the ability of which I was capable to save the teeth of those who sought my services. In some classes of teeth, and in some positions of decay, the permanent separation of the teeth is feasible: as, for instance, in those positions where the decay has not encroached high up on the neck of the teeth, where the enamel is thin, and where it is possible to obtain a firm point of contact. When I read the earnest protests of the Contourists against separation, and was fain to admit their strong argument in favor of their plan—viz., the taking of nature for a guide, and of restoring by art the shape of the tooth, which was ruined by excessive decay—I thought to myself, "Is it possible that the Contourists can be right, and I, with all the thought I have given to this subject, wrong?" I knew that my efforts had been in earnest, and that I had endeavored to do the best I knew how for the patients who employed me; but I am free to confess, after years of experience, that, in the very large majority of-cases, the contour plan is the correct one, and I beg to be here recorded as one who has seen the wisdom of it.

*Dr. Kingsbury, Philadelphia.*—I want to express my hearty endorsement of the paper before us, one of the fullest and most satisfactory papers in my estimation on the subject that it has been my good fortune to listen to, compared with some of the older men of the profession who are present with us to-day, I can but regard myself as quite a young man, a mere student in dental practice. Drs. Atkinson and Dwinelle will leave a rich legacy to the dental profession of America and the world. I was somewhat surprised at the decided and strong position taken by Dr. Arthur some years ago in his practice of filing and leaving such wide spaces between the teeth, yet many of our best practitioners adopted his method, and with others I was led to believe it most beneficial in many cases for the preservation of the natural teeth. While there may be arguments adduced in favor of Dr. Arthur's method of practice, I have long since come to fully recognize the demerits and to practice the other method, that of contour fillings, and this is my present mode of operating in a large proportion of my fillings. Owing to a lack of skill or proper care in packing gold in approximal cavities near the margin of the gum, numerous failures it must be admitted have occurred, and this fact has given rise to one of the strongest arguments in favor of Dr. Arthur's method of filling teeth in such cases. I am of the opinion that in special forms of teeth a marked separation will sometimes better conserve their preservation.

*Dr. S. H. Guilford, Philadelphia*—I am in perfect accord with the sentiments expressed in the paper, and it seems to me that the day of permanent separation of the teeth has gone by. Almost everyone now believes that the best plan of treating these teeth is to separate them well, remove all the decay and weak portions of the teeth; fill thoroughly with gold, contour them, finish well, and then allow them to approximate again.

In order that contour work may be properly done, several things are necessary. First, we must have a sufficiency of room, space in which to operate, and that space is made in one of two ways, either by gradual pressure or by immediate separation at the time of the sitting. For my part, in a majority of cases, I advise immediate separation. It can be done with little or no pain. Before I undertook to use the separator I was somewhat in doubt as to how my patients would like it. I thought there would be a great deal of pain, but I found that was not necessarily so if due care was exercised. I frequently find in making temporary separations, especially for children, that whatever we place between the teeth is liable to be displaced. By means of separators we avoid this, and there is no soreness following the operation.

Varney and Webb could build up gold so as to restore the natural form of the tooth, but the great majority of us are not so skillful as that; consequently, if we can get the aid of some mechanical device we are much benefited.

Dr. Perry spoke well regarding matrices. They play an important part in certain work, and a practitioner to be always ready should have matrices of all the different kinds, for all will come into place on some occasion. One objection urged against their use is that they limit the space for operating and make it difficult to fill the margins perfectly at the cervical wall or lateral border. With regard to limiting space, this is true; but it is no serious objection, and in regard to margins, that depends upon the style of matrix used and the manner of adjusting it. I have always held that the proper matrix to use is one that is slightly flexible, so that when placing gold against the wall, the matrix will yield slightly and allow the gold to go a little beyond the line of contour. The same is true in regard to the cervical margin.

Not a little advantage in the use of the matrix is the little time that is required for trimming after the filling is inserted. Good results can be obtained by the use of matrices in the packing of cylinders, mats, etc., at the cervical border, by having them a little longer than the cavity is deep. Then by placing them against the pulp wall and leaving the other end free to tuck in between the matrix and the edge of the tooth, we hold the first or foundation cylinders perfectly in place, and can mallet them well down and secure perfect marginal contact. When this is done perfectly, having a matrix that is a little elastic, there is no difficulty about obtaining satisfactory results. It requires a little care and skill, but we get better results than we could obtain in any other way.

Regarding the separation of the teeth, I had a little child brought to me a few weeks ago that had some temporary fillings placed between the superior incisors and laterals that had been there since early in the summer. The dentist had separated the teeth and then packed gutta-percha in the spaces to keep the teeth apart. The material pressing upon the gum had also made it very sore. The excessive separation of the teeth had forced the lateral underneath the cuspid that was coming in. In separating teeth for filling we should not have them separated any length of time, for I have seen cases, as Dr. Perry mentioned, where teeth have not returned to their former position for a long time, and sometimes never.

In regard to the dental engine, Dr. Pierce spoke rather discouragingly of its use by beginners. It possesses the possibility of both good and evil. Students must be taught to use it carefully. I agree with Dr. Pierce that it would be a good thing if the dental engine was not used by students until they had spent some time in college. I have always urged students to do without it the first year so as to acquire some dexterity in manipulation, after which they will better understand how to use and control the engine.

*Dr. J. N. Crouse, Chicago.*—I wish to endorse in toto the principles laid down in the paper on contour work. We have now the separators which aid us very much in securing space while we are working, and there is very little excuse for fillings not being contoured for the protection of the gum, the comfort of the patient and the advancement of the operator.

I know that good fillings can be made with sponge gold, but have substituted heavy foil instead, which fills a place for me nothing else can.

I took strong grounds against Dr. Arthur's method when his book came out in favor of contouring, but after condemning it, and seeing others going into separating, I was dragged away from my firm resolution for a little while, but went back to it, being satisfied that contouring gave the very highest results and is true artistic dentistry, affording the greatest amount of comfort to the patient as a masticating apparatus, but requiring a great deal of good judgment, skill and energetic labor.

*Dr. W. H. Dwinelle, New York City*—I always stand up for crystal gold. I may surprise many present when I say that crystal gold is the author of contour filling. It was because of the adhesive principle in crystal gold that contour fillings were made possible. Dr. Watts and myself were the first to discover that absolute gold contained in itself an inherent principle of adhesiveness, and hence the possibility of building gold up in an independent and consolidated form.

I refer to my treatise of "Crystal Gold," published in 1855. In it I advocated the contouring of teeth with gold, and demonstrated it by practical illustrations of actual work. My statements were deemed so extravagant that I was ridiculed and dubbed the Munchausen of the profession. Now some of those who were the most zealous in condemning my system and its possibilities claim to have originated it themselves. At that time I claimed that I originated the system of contouring teeth to their normal forms, and stated that I was willing to give a thousand dollars to any one who would bring proof to the contrary. I respect the offer to-day.

Absolute gold being adhesive, it becomes plastic in our hands; and from this standard of purity, by admixture of slight alloys, we are enabled to make foils cohesive, semi-cohesive and soft or non-cohesive.

Subject passed.

DISCUSSION ON DR. GUILFORD'S PAPER ON VOLUNTARY TOOTH MOVEMENT RESULTING IN EXCESSIVE INTERDENTAL SPACES.

*Dr. W. A. Atkinson, New York City*—I admire the lecturer for the charm of his ideas and the perspicuity of his expression; that was a case of pyorrhœa alveolaris. He said it was diffcult to understand how that tooth moved from the place when it was tied. As to knowing the cause and how to get at it, he smeared it over without the clear analytical discrimination he is capable of exercising.

A patient came to me awhile ago suffering with one of worst cases of pyorrhœa alveolaris I ever saw, without the loss of a tooth, who told me she had not eaten a single comfortable meal in six years. With waxed floss silk the teeth were tied in their proper relations; they were so loose they had to be held carefully in adjusting silk. The right inferior molar had its anterior root two-thirds absorbed, was very loose and probably there was some absorption on the posterior root. I would not extract it because I wanted it to tie to, that each tooth should stand according to a perfect occlusion affected by the use of corundum wheels, I did not attempt to remove all of the lime at that time. Bibulous paper was placed around the pockets and aromatic sulphuric acid injected until the pockets would not drink any more. You could see it flowing into the depths of the sockets. It does not remain in sight very long. Bibulous packs of several folds, smeared with a paste of tannin and glycerine, were placed to prevent the acid from touching the mucous membrame in the mouth. Repeat above and below; above it will be capillarity that will help us; and below we will have gravity too; with a good delicate syringe introduce drops into the pockets until they are full.

That lady came the next day and said: "I have eaten the first meal comfortably in six years."

*Dr. James Truman, Phila.*—I cannot agree with Dr. Atkinson that a case of the kind described is always necessarily pyorrhœa alveolaris. It is well known that calcarious deposits will remain for long periods of time without producing this pathological condition; indeed, I hold that pyorrhœa is not possible with salivary calculus adherent to the teeth. If this were a producing cause, then would the inferior incisors be rarely free from this disease.

In regard to the interdental spaces described by Dr. Guilford, there is no question but that a great wrong is done in the extraction of some teeth, especially the first molars. When these interdental spaces occur, and it must be exceptionable when they do not, an irregularity is produced almost impossible to regulate. What is needed is to understand that all teeth are important and to be preserved; in a word, that extraction is the very last thing to be done.

*Dr. S. H. Guilford, Philadelphia*—I will state that the word "voluntary" was used for want of a better term. Regarding what Dr. Atkinson has said about the formation of pus, it seems to me that if pus had been there (and pyorrhœa means pus), there would have been evidence of it in ten years' time. In all of those cases mentioned to-day and others, I have never found any evidence of pyorrhœa. The inflammation has not extended to suppuration. In regard to a pocket being there, the only pocket we find is caused by the separation of the pericemental membrane from the root, and this is apt to favor the secretion of tartar.

*Dr. Atkinson*—What sort of tissue did you go through when you came to those pockets?

*Dr. Guilford*—No tissue at all; I passed beneath the dental ligaments.

*Dr. Atkinson*—That ligament was gone long before.

*Dr. Isenbrey*—Had these teeth ever been separated to bring them apart? Very often, in my opinion, injudicious separation of the teeth starts that condition of affairs.

*Dr. Guilford*—They had not.

*Dr. Atkinson*—There was from your description of it. The lime does not deposit in connective tissues. There is a little pocket for the lime salts to crystallize in. These are artificial pockets made by a break or fracture of the tissue, so I supposed from your own description you tried to take out that.

*Dr. Guilford*—One of these cases was followed for ten years and yet no pus was discovered.

*Dr. Atkinson*—So was the case I mentioned. The alveolar wall was destroyed so that the gum only hugged the teeth above the necks. Upon proper examination by putting a little cotton in between them, pus corpuscles and microbes in abundance were found.

*Dr. Sudduth*—We only have pus where there is suppuration.

*Dr. Atkinson*—(Interrupting), I said separation.

*Dr. Sudduth*—I did not say that you said suppuration; you, however, did say that it was a case of pyorrhœa alveolaris, which name is only given to that stage of the catarrhal condition that attacks the gums and alveolar process when it has reached the suppurative stage. In regard to the movements of teeth in these positions there can be no question but that they do change their position without any perceptible inflammatory condition; in proof of which, witness the deposits of lime salts—serumal secretions—which are deposited only when inflammation has not progressed very far. Serumal deposits result from a low grade of inflamma. tion, in which case no pus is formed. These cases of Dr. Guilford's are not cases of pyorrhœa alveolaris.

*Dr. Atkinson*—Where do you understand that the space occupied by the consolidated lime salts to be, than except in a pocket in the tissue. Suppose you take the eye, for instance. Is there not a chasm there into which the inspirated lime salts go?

*Dr. Sudduth*—The lime salts are deposited in the inter-cellular protoplasm; there is no actual cavity.

Subject passed.

To be continued.

